# Undernutrition, Host Immunity and Vulnerability to Infection Among Young Children

**DOI:** 10.1097/INF.0000000000002363

**Published:** 2019-08-01

**Authors:** Agnes Gwela, Ezekiel Mupere, James A Berkley, Christina Lancioni

**Affiliations:** * The Childhood Acute Illness & Nutrition (CHAIN) Network, Nairobi; † KEMRI-Wellcome Trust Research Programme, Kilifi, Kenya; ‡ Department of Pediatrics, Makerere University, Kampala, Uganda; § Centre for Tropical Medicine & Global Health, University of Oxford, Oxford, United Kingdom; ¶ Department of Pediatrics, Oregon Health and Science University, Portland, Oregon

**Keywords:** pediatric, immunology, environmental enteric dysfunction, microbiota

Undernutrition contributes to nearly 50% of all annual deaths in children under 5 years. Commonly termed “malnutrition,” undernutrition can have an acute, chronic or acute-on-chronic presentation and refers to children who are underweight for their age or length, too short for their age or deficient in key macro or micronutrients. Undernourished children are more vulnerable to infectious pathogens and more likely to die from infectious diseases; specifically, complications related to diarrhea, pneumonia and measles. Although there has been some progress in reducing childhood undernutrition worldwide, children living in sub-Saharan Africa and Asia continue to bear the largest burden of morbidities and mortality associated with undernutrition. Undernutrition not only impacts growth and vulnerability to severe infection, but chronic undernutrition is highly associated with lifelong cognitive delays. Thus, undernutrition deprives children from thriving to meet their full growth and cognitive potentials, introducing disadvantages that persist into adulthood.

Despite strong evidence of associations between undernutrition, infection and an increased risk of death among young children, the mechanisms driving this vulnerability are not understood. Efforts to tease out causal pathways are hampered by the complex interplay between nutritional status and infection that results in a vicious cycle. In this cycle, infection results in undernutrition due to nutrient loss, reduced uptake and increased energy requirements; while undernutrition drives an increased risk of infection by reducing gut barrier function, modifying the intestinal microbiota, altering regulation of inflammatory adipocytokines and limiting the uptake of key micro and macronutrients. Here, we will explore specific components of this vicious cycle that may compromise host immunity to impact vulnerability to infection among children in low and middle-income countries (LMICs).

## Altered Gut Barrier Function and Chronic Inflammation

The human gut provides an essential barrier against invasion by external pathogens but can also serve as a potential entry point for microbes, pathogen-associated molecular products (PAMPs) and environmental toxins. Many children living in LMICs suffer from environmental enteric dysfunction (EED), a syndrome driven by increased environmental exposure to poor sanitation, intestinal pathogens and subsequent chronic inflammation. A hallmark of EED is increased activation of mucosal immune cells, erosion of epithelial barrier cells, villous atrophy and crypt hyperplasia.^1^ These changes cause inflammation of the small intestine, decreased nutrient absorption, increased intestinal permeability and systemic translocation of immune-stimulatory PAMPs such as lipopolysaccharide. Although the direct impact of EED on host immunity and vulnerability to early childhood infections has not been defined, chronic systematic exposure to lipopolysaccharide has been shown to significantly alter innate cytokine production, the costimulatory capacity of antigen-presenting cells, impair T-cell proliferation and induce cross-tolerance to PAMPs among both HIV-infected and uninfected adults.^2^ Such chronic immune stimulation may result in immunoparalysis that predisposes to recurrent invasive infections. Children living in LMIC where low levels of hygiene persist will have increased exposure to environmental pathogens and PAMPs. Thus, EED-driven immune paralysis is likely a central driver of immune dysfunction resulting in increased vulnerability to both acute and chronic infections during childhood.^3^

## Intestinal Dysbiosis and Host Immunity

The intestinal microbiota, composed of a complex community of bacteria, fungi, archaea and viruses, contributes to a diverse array of biologic functions impacting both host nutritional status and immunity. For example, bacteria living as commensals within the intestines produce short-chain fatty acids, essential vitamins and facilitate mineral absorption. Interactions between the microbiota and host cells are thought to be critical for the maintenance of gut barrier integrity, promotion of mucosal immunity and production of hormones and neurotransmitters that govern metabolism.

Establishment of the intestinal microbiota (or “microbiome,” referring to the DNA of those organisms composing the microbiota) begins during gestation and is strongly influenced by maternal factors, mode of delivery and breast-feeding practices. Despite these influences, a characteristic pattern for acquisition of different components of the intestinal microbiota over the first 3 years of life has been validated in multiple populations, and the concept of a “microbiota-for-age-Z-score” has been introduced.^4^ Moreover, it is apparent that undernutrition is both a consequence of, and/or driver for, intestinal dysbiosis (a term referring to a maladapted or unbalanced microbiota). A study of Malawian children that enrolled well-nourished twin pairs, or discordant twins with one suffering from severe protein malnutrition (termed kwashiorkor) and the other twin being well nourished, dramatically illustrates the relationship between nutrition and host microbiata.^5^ Investigators found that despite provision of nutritional rehabilitation, the microbiota of infants with kwashiorkor were more restricted and “immature” when compared with their well-nourished siblings. When gnotobiotic mice were provided with a protein-poor diet similar to that consumed by Malawian children, and given a fecal transplant from children with kwashiorkor, they rapidly lost weight as compared with animals transplanted with stool from well-nourished twins. Further analysis revealed that animals receiving fecal transplant from children with kwashiorkor had a deficiency in bacteria with anti-inflammatory properties; and although this intestinal dysbiosis improved with refeeding, the improvement was transient. Dissecting the reciprocal relationships between nutrition, the intestinal microbiota and immune function is a critical research priority.

## Metabolism and Cellular Immunity

Adipose tissue is the main storage site for nutrients, serving as a sensor for inadequate energy stores and using secretion of hormones and cytokines (termed “adipokines”) to control both cellular metabolism and immune activity. Thus, undernutrition leading to reduction in adipose tissue volume and stores directly impacts host immune activity.^6^ Leptin is one of the best described adipokines, serving as a critical mediator of glucose and lipid metabolism, angiogenesis, hematopoiesis and innate and adaptive immune function. Nutrient intake leads to a surge in leptin production that stimulates activation, proliferation and production of pro-inflammatory cytokines [interleukin (IL)-6; tumor necrosis factor-α] by monocytes, macrophages, dendritic cells, and Natural Killer cells. Leptin also promotes T-cell activation and development toward pro-inflammatory Th-1 and Th-17 cell subsets. Conversely, adiponectin is an adipokine produced during times of nutrient restriction. It serves to promote activity of the so-called “M2 or alternative” macrophages to encourage secretion of anti-inflammatory cytokines IL-10 and IL-1Rα, limit activation of the pro-inflammatory NK-κB pathway and reduce both T-cell responses and B-cell production. During the periods of nutrient deprivation, diminished production of leptin and increases in adiponectin secretion, combined with alterations in the hypothalamic access driving production of the stress hormones cortisol, restrict the ability of immune cells to generate pro-inflammatory immune responses. Upregulation of glycolytic pathways with restriction of oxidative phosphorylation, referred to as the “Warburg effect,” is required to meet the high metabolic costs of pro-inflammatory effector cells, and cannot occur in this milieu.^7^ Thus, restrictions on glycolysis and glutaminolysis during the periods of nutrient depravation limit T- and B-cell activation and lead to preferential activation of regulatory T cells that rely on alternative energy sources (fatty acid oxidation). In addition, metabolic constraints limit the capacity of pro-inflammatory macrophages and neutrophils to infiltrate the site of infection, phagocytose and kill bacteria through the production of reactive oxygen species. Immune cell proliferation and production of pro-inflammatory cytokines known to be vital to pathogen containment and killing, such as tumor necrosis factor-α, IL-6 and IL-8, are reduced during the periods of starvation, whereas anti-inflammatory cytokines such as IL-10 and IL-33 are increased.^8^ Given the profound restrictions in both innate and adaptive immunity that occur during the periods of nutrient restriction due to altered regulation of adipokines, it is not surprising that undernutrition has a profound impact on vulnerability to and outcomes from a variety of childhood infectious diseases.

## Micronutrient Deficiencies Associated with Infectious Diseases

Casual links between vitamin A deficiency and outcomes from measles are well established, and vitamin A supplementation is critical to the care of children with measles. More controversial is the association between tuberculosis (TB), caused by infection with *Mycobacterium tuberculosis* (Mtb), and low serum levels of vitamin D in children.^9,10^ The active form of vitamin D, 1α,25-dyhydroxyvitamin D3 (referred to as “vitamin D3”), has a broad impact on host immune functions relevant to control of Mtb infection. A variety of immune effector cells express the vitamin D receptor, and vitamin D3 drives the differentiation of monocytes into macrophages and boosts the capacity of these cells to phagocytosis mycobacteria. Vitamin D3 also enhances the production of antimicrobial peptides by host phagocytes and promotes autophagy by Mtb-infected cells; in vitro, these effects led to enhanced inhibition of Mtb growth. Vitamin D3 impacts adaptive immunity by limiting pro-inflammatory Th1 and Th17 responses and promoting production of anti-inflammatory cytokines such as IL-10, IL-4 and transforming growth factor-β. Vitamin D3 also limits proliferation and differentiation of B cells to reduce immunoglobulin secretion. Thus, based on in vitro findings, vitamin D3 has the potential to promote Mtb containment and elimination by components of the innate immune system, while also limiting pro-inflammatory adaptive responses that could exacerbate tissue destruction at the site of disease.^11^

The importance of vitamin D in promoting good outcomes among patients with TB, delivered through exposure to sunlight and cod liver oil, was recognized as early as the 19th century. However, modern trials of vitamin D supplementation as an adjuvant to TB-specific antibiotics have had conflicting results. Moreover, it remains unknown if vitamin D supplemental can reduce the risk of Mtb infection following an exposure. An on-going phase 3 double-blind randomized placebo-controlled trial in Mongolia will address the role of vitamin D3 supplementation in reducing the incidence of latent Mtb infection among school-age children. As infants are uniquely vulnerable to TB and have well-recognized differences in adaptive and regulatory immune functions as compared with older children, determining the impact of vitamin D3 supplementation on prevention of Mtb infection and TB in this age group remains a priority.

## Conclusions and Implications

The consequences of undernutrition on childhood morbidity and mortality resulting from infectious diseases are well recognized. The research community must continue to strive to understand mechanisms that predispose undernourished children to infection, and how infection further compromises both acute and long-term nutritional stores. Longitudinal studies beginning during early infancy and/or pregnancy that untangle complex interactions between nutritional status, host immunity and risk of disease and disease outcomes from specific pathogens should be prioritized. Breaking the vicious cycle between undernutrition and infection will require novel interventions that restore immune homeostasis to promote lifelong host defense from a diversity of pathogens.
